# Determinants of Childhood Immunization Uptake among Socio-Economically Disadvantaged Migrants in East China

**DOI:** 10.3390/ijerph10072845

**Published:** 2013-07-09

**Authors:** Yu Hu, Qian Li, Enfu Chen, Yaping Chen, Xiaohua Qi

**Affiliations:** Institute of Immunization and Prevention, Zhejiang Center for Disease Control and Prevention, Hangzhou 310051, China; E-Mails: qianli@cdc.zj.cn (Q.L.); enfchen@cdc.zj.cn (E.C.); ypchen@cdc.zj.cn (Y.C.); xhqi@cdc.zj.cn (X.Q.)

**Keywords:** immunization, migrants, child health, determinants

## Abstract

*Objective*: To determine the coverage of childhood immunization appropriate for age among socio-economically disadvantaged recent migrants living in East China and to identify the determinants of full immunization uptake among these migrant children. *Methods*: This is a cross-sectional survey of 1,426 migrant mothers with a child aged ≤24 months, who were interviewed with a pretested questionnaire. Various vaccines, migration history and some other social-demographic and income details were collected. Single-level logistic regression analyses were applied to identify the determinants of full immunization status. *Results*: Immunization coverage rates are lower among migrants and even lower among recent migrants. The likelihood of a child receiving full immunization rise with parents’ educational level and the frequency of mother’s utilization of health care. Higher household income also significantly increase the likelihood of full immunization, as dose post-natal visits by a health worker. *Conclusions*: Recent migrant status favours low immunization uptake, particularly in the vulnerability context of alienation and livelihood insecurity. Services must be delivered with a focus on recent migrants. Investments are needed in education, socio-economic development and secure livelihoods to improve and sustain equitable health care services.

## 1. Introduction

An estimated 2.7 million children die annually from vaccine-preventable diseases, of which 40% occur in the Asia Pacific region [1]. Immunization has been shown to be one of the most cost-effective health interventions worldwide, through which a number of serious childhood diseases have been successfully prevented or eradicated. Despite this, the implementation of immunization programmes varies greatly across different communities and approximately 34 million children worldwide do not have access to any immunization services [2,3]. China’s immunization program was established in 1978. In 2005, a decision of the central government has made that all these vaccines would be purchased by the government and provided to all children (including migrant children) free of charge, without any extra service fees. Despite China’s great progress across a range of health indicators in the past decades [4], measles incidence, though fluctuating in the past two decades, has remained high. Similar to other countries [5], increasing population mobility coupled with low routine vaccination coverage of migrants has been identified as one of the key contributors to measles outbreaks.

Disparities in health conditions and health care utilization are evident between local residents and migrants and this phenomenon also exists between recent and long-term migrants, who are more marginalized and vulnerable until they have adapted to the social and cultural norms of a new place. To understand the use of health care services, determinants models are generally applied [6]. The vulnerability of migrants, particularly of recent migrants, is obvious in terms of livelihood insecurity, negligence and alienation in the new sociocultural environment. It leads to less control over available resources that are meant for all members of communities including migrants. We hypothesize that socio-economically disadvantaged migrants, particularly recent migrants, are more likely to forego health care than the general population and settled migrants.

We anticipate that the disparities and inequities within areas persist in immunization coverage and that the socio-economically disadvantaged, particularly those who migrated recently from rural villages, are more vulnerable and may contribute to the lower uptake of immunizations. Our study aimed to: (1) report the reception of various vaccines appropriate for age among the socioeconomically disadvantaged migrants living in Yiwu, China and (2) to determine individual-, household- and health care system-level determinants associated with full immunization uptake.

## 2. Materials and Methods

### 2.1. Study Area and Study Groups

The study was carried out in Yiwu city, located in Eastern China. Yiwu has a total area of 1,105.5 km^2^ and is divided into 716 administrative villages. The total population of the district is 2.17 million inhabitants and its local economy is based on small commodity trade and vibrant free markets. Yiwu’s rapid development had attracted some 1 million migrants, particularly from rural areas of China, since 1992 when the vibrant free markets were established.

Target population in our survey was made up of migrant children under two years and their mothers. Children who were not local residents were included if they had lived in the surveyed areas continuously for 1 month or more at the time they were interviewed. There were two reasons for this choice. The first reason was that primary immunization was regarded as completion of all the required doses at 12 months of age according to the Ministry of Health’s (MoH) guideline on evaluation of immunization coverage [6]. The second reason was that migrant children who had lived in their catchment areas continuously for 1 month or more had to be identified and immunized by health centers in Yiwu.

We categorized migrants into two groups: recent migrants and settled migrants. Recent migrants were those who moved to Yiwu within the last 3 years, and settled migrants were those who had been residing in Yiwu for at least 3 years. Both groups were mainly from middle/western areas of China, particularly Anhui, Henan and Guizhou Provinces, and their distribution in terms of place of origin, ethnic, social class and religion was similar.

### 2.2. Childhood Primary Immunization Schedule

Health centers in Yiwu adopt the primary vaccination schedule recommended by the MoH [7], which stipulates that infants should be vaccinated with the following vaccines within the first 12 months: a birth dose of Bacille Calmette-Guérin (BCG), three doses of oral poliovirus vaccine (OPV), three doses of diphtheria-tetanus-pertussis (DTP) vaccine, three doses of hepatitis B vaccine (HepB), one dose of measles-rubella combined vaccine (MRV), and one dose of Japanese Encephalitis Vaccine (JEV) (Table 1).

**Table 1:** Recommended childhood primary immunization schedule in China.

	Age								
	Birth	1 m	2 m	3 m	4 m	5 m	6 m	8 m	
BCG	Dose1								
HepB	Dose1	Dose2					Dose3		
OPV			Dose1	Dose2	Dose3				
DPT				Dose1	Dose2	Dose3			
MRV								Dose1	
JEV								Dose1	

### 2.3. Sampling

Initially, several resettlement colonies where migrants reside were identified by the original data issued by Bureau of Statistics of Yiwu city. Finally, a total of 56 resettlement colonies were selected. Recent and settled migrants were selected from resettlement colonies. The sampling method was based on the World Health Organization (WHO)-advocated cluster sampling technique [8]. All 56 resettlement colonies selected served as clusters.

Primary immunization coverage rates for the studied five vaccines of children under 2 years was assumed to be 80% and the desired precision was ±3%. We assumed a design effect of 2 and obtained required number of surveyed children per cluster for variable numbers of clusters through the table recommended by WHO manual (see Appendix). Twenty-five children per cluster for 56 clusters were finally determined as our sample size. Interviewers obtained a household list from community authorities in each selected resettlement colony and randomly selected one household (using random numbers) as the first family of an eligible child to be interviewed, and continued by choosing each subsequent household located to the right of the previous one until 25 eligible children per each birth cohort were interviewed. Only one child per household was selected to avoid clustering. When two or more eligible children were in the same household, the youngest child was selected based on the WHO manual.

### 2.4. Data Collection

Demographic and socio-economic details, migration history, the status of immunization received and mother’s use of health care services were elicited through a face to face interviewer-administered questionnaire. The immunization status of the child was determined from the immunization card, and in the absence of immunization cards, mothers were asked to recall whether the child had received different vaccines (including the number of doses for each). Separate questions were asked to extract information on the each age-appropriate vaccine to be received. Before data collection, a parent or legal guardian of each child enrolled provided signed informed consent. In each household surveyed the informed consent form was discussed with the parents or legal representatives of the child, and signed by one of them once there was a decision to participate. The survey was considered to involve no biological specimen collection or vaccination, therefore, it did not require Zhejiang Provincial CDC institutional review board approval according to the MoH’s regulation of ethical review of biomedical research involving humans [9].

### 2.5. Measurement

Two outcome measures were considered: the likelihood of a child over 12 months of age having received: (1) full immunization against seven vaccine preventable diseases (VPDs) (one dose of BCG; three doses each of DPT and OPV; one doses of JEV; one dose of MRV), from now on referred to as full immunization against seven VPDs, and (2) full immunization against eight VPDs (which includes three doses each of HepB in addition to the abovementioned vaccines), from here onwards referred to as full immunization against eight VPDs. Individual-level independent variables were gender and birth order of the child. The household-level characteristics were mother’s age, educational status and mother’s occupational status (working to earn, not working to earn); father’s educational status, Occupation of the head of the household, household income per month (in Chinese Yuan, 1 CNY = US$ 0.16), ethnic (Han, minority ethnic), size of the household, and migration status (recent migrants, settled migrants). Mother’s health care utilization was assessed by her attendance of antenatal care (ANC) and place of delivery (home, hospital). The system-level variable was post-natal visits by a health worker.

### 2.6. Data Analysis

Age-appropriate vaccination was taken as the proportion of children who received particular vaccines appropriate for their age to the total number of children in that particular age group and 95% confidence intervals (*CIs*) were also calculated. To examine the association of several exposure (independent) variables on full immunization, children >12 months of age were considered for analysis. We employed the single-level logistic regression model to analyze the variables associated with full immunization against seven/eight VPDs (two outcome variables mentioned earlier) for children >12 months, and we presented the adjusted odds ratio (AOR) with 95% *CI.* Each of the dependent variable was dichotomized as “fully immunized/not fully immunized”, and the single-level logistic regression analyses were carried out separately. Initially, each independent variable was regressed against each dependent variable. Since we used a *p* value of <0.1 as a screening criterion for variable selection in this study, those variables with a minimum *p* value of 0.1 were considered for logistic regression analysis. The use of a more usual *p* value (such as 0.05) often fails to identify variables known to be important, while the use of a higher *p* value has the disadvantage of including variables that are of questionable importance. Single-level logistic regression analyses were carried out by backward likelihood ratio method. All the analysis above applied Statistics Package for Social Science (SPSS) software, version 13. 0 (SPSS Inc, Chicago, IL, USA).

## 3. Results

### 3.1. Socio-Demographic Characteristics

Of the 1,471 mothers contacted, seventeen (1.2%) mothers refused to participate in the survey and the data of another 28 mothers were incomplete. Thus, the final analysis was based on data from 1,426 mothers (all of their children had immunization cards), of which 847 (59.4%) mothers were recent migrants.

Of all 1,426 children, 50.9% were ≤12 months at the time of survey, and 50.4% were male. 86% of the surveyed children’s ethnic were Han, which is the majority ethnic in China. 56.7% of the households interviewed had only one child. 50.2% of the mothers and 46.4% of the fathers only received primary school or less. Of all the surveyed mothers, 68.4% were 20 to 30 years old and 73.8% were housewives. Regarding the occupation of the head of the household (all were men, except for fifteen widows), the majority were unskilled workers and only 6.9% of them earned more than CNY 4,500 (US$ 725) per month. Around 90% of the children were delivered in hospital. Only 46.2% of the mothers had received ANC and 57.9% got a post-natal visit by a health worker.

In bivariate analyses, significant differences in the distribution of immigrant status were noted by the child’s place of delivery, post-natal visits by health worker and ANC. The primary caregiver’s characteristics, including mothers’ age, educational level of parents, occupation of the mother, and household income also significantly influenced the immigrant status.

### 3.2. Immunization Appropriate for Age

Table 2 presents the details on the reception of various vaccines appropriate for age applied a birth cohort analysis. Around 50% of the children had received HepB at birth, while only 32.6% were given BCG at birth. Migrants’ use of immunization services had a remarkable increase at one month old, but it had fallen considerably as time went by, especially among the recent migrants. Only 42.6% of recent migrants’ and 51.9% of settled migrants’ children received MRV at eight months. Full series immunization coverage for DPT, OPV and HepB among migrants were 57.6%, 64.0% and 52.2%, respectively. Of 410 recent migrant children >12 months of age, the proportion of fully immunized against seven VPDs and fully immunized against eight VPDs were 63.9% (95% *CI*: 59.2%–68.6%), 57.1% (95% *CI*: 52.2%–62.0%), respectively. Of 290 recent migrant children >12 months of age, the proportion of fully immunized against seven VPDs and fully immunized against eight VPDs were 73.4% (95% *CI*: 68.3%–78.5%), 57.1% (95% *CI*: 64.7%–75.3%), respectively.

**Table 2:** Reception of various vaccines appropriate for age among the children of recent and settled migrants.

Vaccines appropriate for age	Sample size (Recent migrants; Settled migrants)	Recent migrants	Settled migrants
		% (95% *CI*)	% (95% *CI*)
Vaccines to be received at birth	847; 579		
*BCG*		26.7 (23.7–29.7)	41.3 (37.3–45.3)
*HepB_1_ (within 24 h)*		35.1 (32.1–38.1)	72.0 (68.4–75.6)
Vaccines to be received at 1 month	796; 527		
*HepB_2_*		59.3 (56.0–62.6)	70.8 (66.9–74.7)
Vaccines to be received at 2 months	752; 503		
*OPV_1_*		55.1 (51.5–58.7)	65.2 (61.0–69.4)
Vaccines to be received at 3 months	704; 471		
*OPV_2_*		51.7 (48.0–55.4)	61.8 (57.4–66.2)
*DPT_1_*		53.8 (50.1–57.5)	63.3 (58.9–67.7)
Vaccines to be received at 4 months	659; 452		
*OPV_3_*		49.8 (46.0–53.6)	58.4 (53.9–62.9)
*DPT_2_*		47.6 (43.8–51.4)	55.3 (50.7–59.9)
Vaccines to be received at 5 months	619; 433		
*DPT_3_*		49.3 (45.4–53.2)	57.0 (52.3–61.7)
Vaccines to be received at 6 months	587; 412		
*HepB_3_*		52.1 (48.0–56.2)	60.1 (55.4–64.8)
Vaccines to be received at 8 months	516; 364		
*MRV*		42.6 (38.3–46.9)	51.9 (46.8–57.0)
*JEV*		42.1 (37.8–46.4)	50.5 (45.4–55.6)
Various vaccines to be received by 6 months	587; 412		
*Three doses of DPT*		55.0 (51.9–59.1)	61.2 (56.5–65.9)
*Three doses OPV*		61.2 (57.3–65.1)	68.0 (63.5–72.5)
*Three doses of HepB*		48.4 (44.2–52.6)	57.5 (52.7–62.3)
Various vaccines to be received by 12 months/1 year of age	410; 290		
*Fully immunized against seven VPDs*		63.9 (59.2–68.6)	73.4 (68.3–78.5)
*Fully immunized against eight VPDs*		57.1 (52.2–62.0)	70.0 (64.7–75.3)
Did not receive any vaccine	847; 579	13.2 (10.9–15.5)	8.8 (6.5–11.1)

### 3.3. Determinants of Full Immunization Uptake

Compared with fully immunized against seven VPDs, the proportion of fully immunized against eight VPDs fell 3.4 units in settled migrant children and that fell 6.8 units among recent migrant children. There were still 11% migrant children who received no vaccines at all.

Of 1,426 children surveyed, 700 children were involved in the determinants analysis as they all were >12 months old. Children of recent migrants were at risk of not receiving full immunization. Compared to recent migrant children, settled migrant children had higher AORs of being fully immunized against seven VPDs (AOR = 1.61, 95% *CI* = 1.12–3.43) and against eight VPDs (AOR = 2.19, 95% *CI* = 1.55–5.38). Other variables such as children’s ethnic, mother’s educational status, mother’s occupation status were significantly associated with the full immunization against seven VPDs (Pearson χ^2^ = 11.503 and *p* = 0.175 for regression model), as were post-natal visits by health worker.

Children’s ethnicity and place of delivery, mother’s educational status and occupation status, household income, as well as mother’s utilization of ANC and post-natal visits by health worker, were significantly associated with full immunization against eight VPDs(Pearson χ^2^ = 5.574 and *p* = 0.695 for regression model). See Table 3.

**Table 3:** Results of single logistic regressions showing the AORs of a child receiving full immunization by various variables.

Variable	Full immunization against seven VPDs ^1^			Full immunization against eight VPDs ^2^		
	n	AOR ^#^ (95% *CI*)	*p*	n	AOR (95% *CI*)	*p*
*Ethnic*			<0.01			<0.01
minority ethnic	33	Reference		29	Reference	
han	442	2.71 (1.04–4.22)		398	3.24 (1.27–4.19)	
*Mother’s educational status*			<0.001			<0.001
Primary school or less	207	Reference		181	Reference	
junior high school	184	1.47 (0.82–3.46)		169	2.14 (1.17–5.26)	
senior high school or above	84	2.46 (1.32–5.11)		77	2.86 (1.61–5.94)	
*Occupation of the mother*			<0.001			<0.001
housewife	371	Reference		332	Reference	
Working	104	2.47 (1.35–4.88)		95	2.71 (1.83–5.24)	
*Household income per month (CNY)*			>0.05			<0.01
<1,500	83	Reference		61	Reference	
1,500–3,000	203	0.82 (0.31–1.23)		196	1.63 (1.04–2.71)	
3,001–4,500	141	0.94 (0.44–1.65)		135	2.27 (1.61–3.54)	
≥4,500	48	1.21 (0.67–1.82)		43	2.81 (1.35–4.92)	
*Place of delivery*			>0.05			<0.01
home	18	Reference		2	Reference	
hospital	457	1.57 (0.84–3.18)		425	3.22 (1.86–6.14)	
*Post-natal visits by health worker*			<0.001			0.00
did not visited	166	Reference		133	Reference	
visited	309	2.71 (1.59–3.62)		294	3.19 (1.61–5.17)	
*Reception of ANC*			>0.05			<0.01
no visits	152	Reference		128	Reference	
1 or 2 visits	235	0.75 (0.16–2.27)		218	1.78 (1.36–4.09)	
≥3 visits	88	1.33 (0.54–2.16)		81	2.56 (1.04–4.82)	
*Migration status*			<0.001			0.00
Recent migrants	255	Reference		213	Reference	
Settled migrants	220	1.61 (1.12–3.43)		214	2.19 (1.55–5.38)	

**^#^**: adjusted odds ratio; ^1^: Pearson χ^2^ = 11.503 and *p* = 0.175 for regression model; ^2^: Pearson χ^2^ = 5.574 and *p* = 0.695 for regression model.

## 4. Discussion

Our study presents the first exploration of the barriers to immunization among migrant children living in Yiwu city, China, from the perspective of the parents. It is important to involve the migrant parents in the development of the immunization program as they are key to deciding whether to immunize their children. Apart from this, identifying perceived barriers also identifies opportunities for developing an acceptable and accessible immunization programme.

The immunization patterns among migrants reveal that a large proportion of migrant children, particularly recently migrated children, do not receive a full course of immunization. Recent migrants’ children particularly are at risk of not being fully immunized, and it seems that recent migrants never replicate the patterns of health service use of residents or even settled migrants. Although the great majority of migrants have contact with the health centers when the child is less than 2 months of age, however, the immunization coverage rates fall noticeably because of dropout. The causes need further exploration, but we attribute high dropout rate to problems in demand for vaccinations, client satisfaction with services, and the ability of the immunization program to provide those services. Similarly, the MRV and JEV coverage rates for migrant children is lower than other vaccines due to a much longer time gap, in which the mothers may forget to return back. The OPV coverage rate is also suboptimal, though the OPV campaign is frequently implemented in Yiwu city. Compared with HepB1, the BCG coverage rate was very low at birth. The main reason for this were most of the obstetrical hospital in Yiwu did not provide the BCG immunization and people always concerned about the adverse event after BCG vaccination. As uptake rate declines steadily following the birth, we suggested Yiwu government should take actions as followed. First, immunization sessions should be scheduled to be convenient for parents, e.g., health facility managers should assess their facilities’ immunization schedules at least once a week and change them if necessary to reflect the current needs of the community. Second, local health workers should play an important role in increasing awareness and providing information to the migrant caregivers. Despite availability of immunization services in Yiwu, which is the most developed city in China, recent migrants’ children were not adequately immunized. The main reasons is that the developed city’s advantage (in terms of improved child health services) is offset by determinants such as migrants’ lack of social networks, the poor status of economy and the disruption of utilization caused by migration itself.

Recent migrants’ children are less adequately vaccinated than settled migrants’ children. Our study probably reflects that recent and settled migrants live in the same areas, where the availability of health services is equal, but awareness of and access to the health services may differ. It mainly depends on the duration time in Yiwu. In the light of these findings, we assume that the newly migrated may face with the challenge to survive in an environment with a high cost of living and the difficulty of adapting to a new sociocultural environment, while settled migrants, who are familiar with the city and enjoy some social support, are better able to avail themselves of health services [10,11,12]. Thus, migration status may be responsible for lower uptake of health care services. Migrant adaptation may play a role as integration into the new society progresses and acculturation is associated with other public health service (such as ANC/post-natal visits) as it was reported in a previous study [13].

We do not find any difference in immunization coverage by gender of the child, number of children in the family, and mother’s age, consistent with the results of other studies [14,15] on migrant’s immunization. Similarly, our observations agree with those of some studies: for example, increasing education level of the parents, especially for mothers can improve the fully immunization coverage among migrant children. It may be due to a better understanding of EPI policy and awareness of children’s health among the caregivers with a higher education level. We also found mothers with a better socio-economic status, such as having occupations and a stable income may improve the fully immunization coverage. It means that a stable occupation with a good income frees the household from the struggle of finding work to survive.

In our study, children delivered at health facilities were more likely to be fully vaccinated than children delivered at home. This finding was similar with the study done in Mozambique [16] in which children delivered at home were less likely to complete immunization. The explanation related to this may be that, mothers who give birth at health centers/hospitals are closer to the health service or get more information on immunization, and most of the time the first dose of vaccination is given just after birth. Besides its relation with the place of delivery, our study highlights the importance of post-natal care in communities. It indicated that uptake of prenatal and post-natal care services increases the chances that mothers will access immunization services. The higher odds of receiving full immunization associated with post-natal visits by a health worker seem to indicate that the system’s features and functioning influence people’s health-seeking behaviour. Another reason for some several individual-, household- and system-level factors having a higher odds of fully immunization against eight VPDs than seven VPDs was the immunization coverage for HepB was lower than other vaccine. It may be due that when written immunization history was missing during migration, the child would be regarded as unvaccinated.

We found that ANC follow up was also related with complete immunization coverage which was consistent with the study done in India [17] and Bangladesh [18]. In our study, ANC follow-up gave increased odds of receiving full immunization against eight VPDs, but not seven VPDs, perhaps because mothers who were not delivered in hospital seldom had adequate access to ANC services. Provision of services in the community may be masking the influence of hospital deliveries.

## 5. Advantages and Limitations

Our study shows the need to develop health care services specifically for migrant communities by considering their sociocultural and economic status, and emphasize the importance of undertaking comprehensive health care system research among migrant communities in China. Our findings are important for the Chinese health care system as well as other developed cities in East China. China’s migration is increasing and its characteristics are generalizable, as migrants everywhere share the vulnerability resulting from alienation and livelihood insecurity.

A limitation of the study is its retrospective reporting, which involves reporting bias and thus impacts the reliability of data. We did not collect detailed data on caregivers’ knowledge, attitude and practice toward immunization, thus we can’t asses the association between caregivers’ awareness and immunization. Also, we did not collect data on health centres’ outreach, supply and other manpower, infrastructure-related issues and thus cannot draw any conclusions on the health care provision system. Despite these limitations, the study has methodological strengths, such as the scientifically drawn sample covering two groups of migrants: one that has reached a level of adaptation and another that is at the beginning of adjusting to a new environment.

## 6. Conclusions

The children of migrants, particular of recent migrants, are at risk of not being fully immunized due to the livelihood insecurity and alienation of their families. Utilization of health services, such as ANC and post-natal visits, leads to significantly increased immunization and the likelihood of a child receiving full immunization. Hence, making the system responsive and effective particularly to vulnerable, socio-economically disadvantaged migrants would help in achieving full immunization coverage. Providing secure livelihoods and equitable services are also important for improving and sustaining full utilization of immunization services.

## Appendix A

**Table:** The number of children per cluster sampled for variable numbers of clusters. (desired precision is ±3%)

Desired Precision ±3%		Expected coverage									
		50%	55%	60%	65%	70%	75%	80%	85%	90%	95%
Number of clusters	20	107	106	103	98	90	81	69	56	39	21
	21	102	101	98	93	86	77	66	52	37	20
	22	98	97	94	89	82	73	63	50	35	19
	23	93	92	90	85	78	70	60	48	34	18
	24	89	89	86	81	75	67	57	46	33	17
	25	86	85	82	78	72	65	55	44	31	17
	26	83	82	79	75	69	62	53	42	30	16
	27	80	79	76	72	67	60	51	41	29	16
	28	77	76	74	70	65	58	49	39	28	15
	29	74	73	71	68	62	56	48	38	27	14
	30	72	71	69	65	60	54	46	37	26	14
	31	69	69	67	63	58	52	45	36	25	14
	32	67	67	65	61	57	51	43	35	25	13
	33	65	65	63	59	55	49	42	34	24	13
	34	63	63	61	58	53	48	41	33	23	12
	35	62	62	59	56	52	46	40	32	22	12
	36	60	59	57	54	50	45	38	31	22	12
	37	58	58	56	53	49	44	37	30	21	11
	38	57	56	54	52	48	43	36	29	21	11
	39	55	55	53	50	46	42	36	28	20	11
	40	54	53	52	49	45	41	35	28	20	11
	41	53	52	50	48	44	40	34	27	19	10
	42	51	51	49	47	43	39	33	26	19	10
	43	50	50	48	46	42	38	32	26	18	10
	44	49	49	47	45	41	37	32	25	18	10
	45	48	47	46	44	40	36	31	25	18	10
	46	47	46	45	43	39	35	30	24	17	9
	47	46	45	44	42	39	35	30	24	17	9
	48	45	45	43	41	38	34	29	23	17	9
	49	44	44	42	40	37	33	28	23	16	9
	50	43	43	41	39	36	33	28	22	16	9
	51	42	42	41	39	36	32	27	22	16	8
	52	42	41	40	38	35	31	27	21	15	8
	53	41	40	39	37	34	31	26	21	15	8
	54	40	40	38	36	34	30	26	21	15	8
	55	39	39	38	36	33	30	25	20	14	8
	56	39	38	37	35	33	29	25	20	14	8
	57	38	38	36	35	32	29	24	20	14	8
	58	37	37	36	34	31	28	24	19	14	7
	59	37	36	35	33	31	28	24	19	14	7
	60	36	36	35	33	30	27	23	19	13	7
	61	36	35	34	32	30	27	23	18	13	7
	62	35	35	34	32	29	26	23	18	13	7
	63	34	34	33	31	29	26	22	18	13	7
	64	34	34	33	31	29	26	22	18	13	7
	65	33	33	32	30	28	25	22	17	12	7
	66	33	33	32	30	28	25	21	17	12	7
	67	32	32	31	30	27	24	21	17	12	7
	68	32	32	31	29	27	24	21	17	12	7
	69	31	31	30	29	26	24	20	16	12	7
	70	31	31	30	28	26	23	20	16	11	7

## References

[1] United Nations Children’s Fund The State of Asia-Pacific’s Children 2008. www.unicef.org/publications/files/SOAPC_2008_080408.pdf.

[2] Lee J.W. (2003). Child survival: A global health challenge. Lancet.

[3] The World Bank World Development Report 2009: Reshaping Economic Geography. siteresources.worldbank.org/INTWDRS/Resources/477365-1327525347307/8392086-1327528510568/WDR09_01_Overviewweb.pdf.

[4] World Health Statistics 2010. www.who.int/whosis/whostat/ENWHS10Full.pdf.

[5] Yaméogo K.R., Perry R.T., Yaméogo A., Kambiré C., Kondé M.K., Nshimirimana D., Kezaala R., Hersh B.S., Cairns K.L., Strebel P. (2004). Migration as a risk factor for measles after a mass vaccination campaign, Burkina Faso, 2002. Int. J. Epidemiol..

[6] Obrist B., Iteba N., Lengeler C., Makemba A., Mshana C., Nathan R., Alba S., Dillip A., Hetzel M.W., Mayumana I. (2007). Access to health care in contexts of livelihood insecurity: A framework for analysis and action. PloS Med..

[7] Technological Standards for Expanded Program on Immunization, 2005. www.chinacdc.cn/n272442/n272530/n272712/appendix/20051010142614586.doc.

[8] (2005). Immunization Coverage Cluster Survey-Reference Manual.

[9] Regulation of Ethical Review of Biomedical Research Involving Humans, 2007. www.chain.net.cn/document/20070607182957828540.pdf.

[10] Antai D. (2010). Migration and child immunization in Nigeria: Individual- and community-level contexts. BMC Public Health.

[11] Kidane T., Yigzaw A., Sahilemariam Y., Frederick M., Henry W. (2008). National EPI coverage survey report. Ethiop. J. Health Dev..

[12] Liu D.W., Sun M.P., Liu W.X., Fan C.Y., Lu L. (2007). Comparative study on immunization coverage rates of nine vaccines between local and floating children. Chin. J. Vaccine. Immun..

[13] Do M.M., Hotchkiss D.D. (2013). Relationships between antenatal and postnatal care and post-partum modern contraceptive use: Evidence from population surveys in Kenya and Zambia. BMC Health Serv. Res..

[14] Ibnouf A.H., Van den Borne H.W., Maarse J.M. (2007). Factors influencing immunisation coverage among children under five years of age in Khartoum State, Sudan. S. Afr. Fam. Pract..

[15] Sarab K., Abedalrahman A.R., Sarhat R., Fred N. (2008). Factors predicting immunization coverage in Tikrit city. Middle East J. Fam. Med..

[16] Jani J.V., De Schacht C., Jani I.V., Bjune G. (2008). Risk factors for incomplete vaccination and missed opportunity for immunization in rural Mozambique. BMC Public Health.

[17] Partha D., Bhattacharya B.N. (2002). Determinants of child immunization in four less-developed states of North India. J. Child Health Care.

[18] Mosiur R., Sarker O.N. (2010). Factors affecting acceptance of complete immunization coverage of children under five years in rural Bangladesh. Salud. Publica. Mex..

